# Comprehensive Genomic Survey, Structural Classification, and Expression Analysis of WRKY Transcription Factor  Family in *Rhododendron simsii*

**DOI:** 10.3390/plants11212967

**Published:** 2022-11-03

**Authors:** Ziyun Wan, Xueqin Li, Hefeng Cheng, Jing Zhang, Yujia Chen, Yanxia Xu, Songheng Jin

**Affiliations:** 1 Jiyang College, Zhejiang A&F University, Zhuji 311800, China; 2 School of Life Science and Health, Huzhou College, Huzhou 313000, China

**Keywords:** *Rhododendron*, WRKY family, transcription factor, bioinformatics, gene expression, hormone, abiotic stress

## Abstract

(1) *Rhododendron* is one of the top ten traditional flowers in China, with both high ornamental and economic values. However, with the change of the environment, *Rhododendron* suffers from various biological stresses. The WRKY transcription factor is a member of the most crucial transcription factor families, which plays an essential regulatory role in a variety of physiological processes and developmental stresses. (2) In this study, 57 RsWRKYs were identified using genome data and found to be randomly distributed on 13 chromosomes. Based on gene structure and phylogenetic relationships, 57 proteins were divided into three groups: I, II, and III. Multiple alignments of RsWRKYs with *Arabidopsis thaliana* homologous genes revealed that WRKY domains in different groups had different conserved sites. RsWRKYs have a highly conserved domain, WRKYGQK, with three variants, WRKYGKK, WRKYGEK, and WRKYGRK. Furthermore, cis-acting elements analysis revealed that all of the RsWRKYs had stress and plant hormone cis-elements, with figures varying by group. Finally, the expression patterns of nine *WRKY* genes treated with gibberellin acid (GA), methyl jasmonate (MeJA), heat, and drought in *Rhododendron* were also measured using quantitative real-time PCR (qRT-PCR). The results showed that the expression levels of the majority of *RsWRKY* genes changed in response to multiple phytohormones and abiotic stressors. (3) This current study establishes a theoretical basis for future studies on the response of RsWRKY transcription factors to various hormone and abiotic stresses as well as a significant foundation for the breeding of new stress-tolerant *Rhododendron* varieties.

## 1. Introduction

The WRKY gene family is the largest transcription factor family in higher plants [1]. The WRKY protein is distinguished by the presence of a highly conserved WRKY DNA binding domain of 60 amino acids and a signature amino acid residue “WRKYGQK” at the N-terminal, followed by a C2H2-type or C2HC-type zinc finger structure [2,3]. Although the majority of the WRKY gene family in plants is relatively conservative, deletion and replacement of N-terminal conserved amino acid residues as well as loss of zinc structure in the C-terminal have been founded in some plants in recent years [4]. WRKY transcription factors are classified into three types based on the number of WRKY-conserved domains and the structure of zinc finger motifs. Group I WRKY proteins typically have two WRKY domains, whereas Group II and Group III proteins only have one WRKY domain and a zinc finger structure C2H2(CX4-5CX22-23HXH) or C2HC(CX7CX23HXC) [5]. The WRKY protein also contains nuclear localization signal peptides (NLS) that regulate target genes. Some WRKY proteins also have a leucine zippers domain that can bind to the W-box element ((C/T) TGAC (T/C)) or the cis-acting element SURE in a promoter to promote or inhibit gene expression [6].

Plants have evolved defense mechanisms to resist varieties of biotic and abiotic stresses throughout their lives. WRKY transcription factors are also crucial regulatory components in plant growth and development, dormancy, lignin biosynthesis, and stress tolerance [7,8]. Identifying and deciphering the key genes that respond to plant abiotic stress could provide the foundation for breeding crops that are resistant to abiotic stresses [9,10]. *SPF1*, the first WRKY-encoding gene, was discovered in *Ipomoea batatas* Lamin 1994 [11], and since then, the *WRKY* gene from *Arabidopsis*, rice, cucumber, apple, peach, and other plants has been gradually cloned [4,12,13,14,15]. In *Arabidopsis*, 80% of *WRKY* genes respond to bacterial infection. *AtWRKY25* and *AtWRKY33* regulate salt tolerance through interactions with upstream and downstream target genes [16,17]. Overexpression *OsWRKY30* in rice increased tolerance to rice fungus *Rhizoctonia solani* and fungus *Magnaporthe grisea* [18,19]. Previous studies have also shown that *HvWRKY38*, *OsWRKY12*, *TaWRKY19*, and *TaWRKY2* provide resistance under drought stress [20,21,22]. Furthermore, increasing studies have documented that WRKY proteins are involved in signal transduction processes mediated by plant hormones such as salicylic acid (SA), jasmonic acid [23], and others. For example, *AtWRKY50* and *AtWRKY51* promoted SA biosynthesis in *Arabidopsis* [24]; and JA treatment increased the expression of *AtWRKY17* and *AtWRKY33* [25]. Therefore, in this study, we analyzed the expression of *RsWRKY* genes in response to GA and JA hormones to learn more about their role in the resistance to abiotic stress in *Rhododendron*. It is known that the WRKY family in *Arabidopsis* consists of 72 members, while the WRKY family in rice consists of 109 members. The expansion of genes is one of the leading causes of gene family growth. The Group I and II WRKY families appeared before monocot and dicot differentiation, while the Group III *WRKY* genes appeared later. Some studies have shown that gene expression patterns among different copies of genes are also dissimilar after gene duplication, but the specific mechanism is still unclear [26]. 

*R. simsii* is an essential ornamental plant in Ericaceae; a total of 7 subgenera and 720 species of wild *Rhododendrons* were found in China [27]. To screen *Rhododendron* varieties with strong drought resistance, 36 varieties of *Rhododendron* were selected to study the changes of physicochemical characteristics in *Rhododendron* leaves under simulated drought stress by PEG-6000 [28]. Since soil pH, moisture, high temperature, and resistance to insects are difficulties in *Rhododendron* cultivation. Identifying the functions of *RsWRKY* genes plays a crucial significance in *Rhododendron*. However, the detailed information of the WRKY family in *R. simsii* and their effect under several abiotic stresses was still unclear. In the present study, *WRKY* genes from *R. simsii* were identified, and it may give a novel insight into studying the mechanism of *Rhododendron’s* coping with abiotic stress.

## 2. Results

### 2.1. Identification and Physicochemical Properties Analysis of RsWRKY Gene Family Members 

Sixty-three potential candidate genes were searched in NCBI by BLASTX to predict conserved domains, reconfirmed by PFAM and SMART, and four genes without a complete WRKY domain were eliminated. Moreover, 2 (*RhsimUnG0199600*, *Rhsim10G0148800*) of these 59 were repetitive sequences, which were manually removed. The remaining 57 *RsWRKYs* were identified and designated as *RsWRKY1–RsWRKY57*. Detailed information about *RsWRKY* genes is shown in Table S1. Proteins encoded by 57 *RsWRKY* genes contained 115 (RsWRKY40) to 1661 (RsWRKY43) amino acids, with an average of 405 amino acids. Their predicted molecular weight varied from 12866.11 (RsWRKY40) to 190287.09 (RsWRKY43) Da, and the isoelectric point ranged from 4.94 (RsWRKY9) to 10.12 (RsWRKY32). Furthermore, the threshold values of the aliphatic index varied from RsWRKY17 to RsWRKY56. The grand average of hydropathicity values of all were negative, indicating that all RsWRKYs were predominantly hydrophilic proteins. Subcellular localization results revealed that WRKY proteins were all distributed in the nucleus.

### 2.2. Chromosomal Location of RsWRKY Gene Family Members

**Figure 1:**
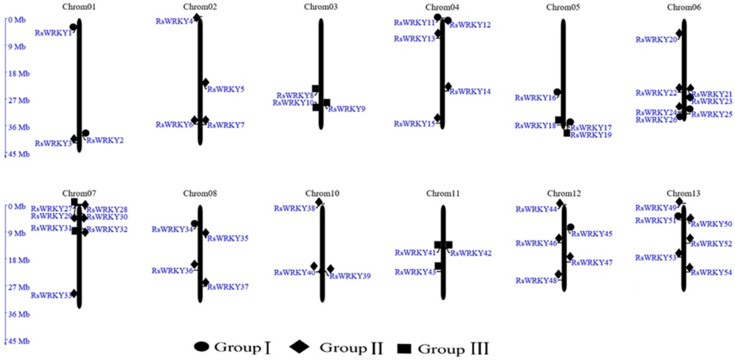
Chromosomal locations of the RsWRKY gene family in *R. simsii*. The 57 putative *WRKY* genes were renamed from *RsWRKY1* to *RsWRK57* according to their chromosomal locations. Three *WRKY* genes that could not be mapped to any chromosome were named *RsWRKY55–RsWRKY57*, respectively.

Chromosomal localization analysis revealed that 57 of the candidate genes were unevenly distributed across all 13 chromosomes of the *R. simsii* genome, with the exception of chromosome 9. The remaining three (*RsWRKY55–RsWRKY57*), meanwhile, could not be mapped to any chromosomes (Figure 1). From the perspective of a single chromosome, the highest number of genes (seven) were located onchromosomes 6 and 7. Notably, three *WRKY* genes were mapped on each of chromosomes 1, 3, 10, and 11.

### 2.3. Phylogenetic Analysis and Classification of RsWRKY Proteins

**Figure 2:**
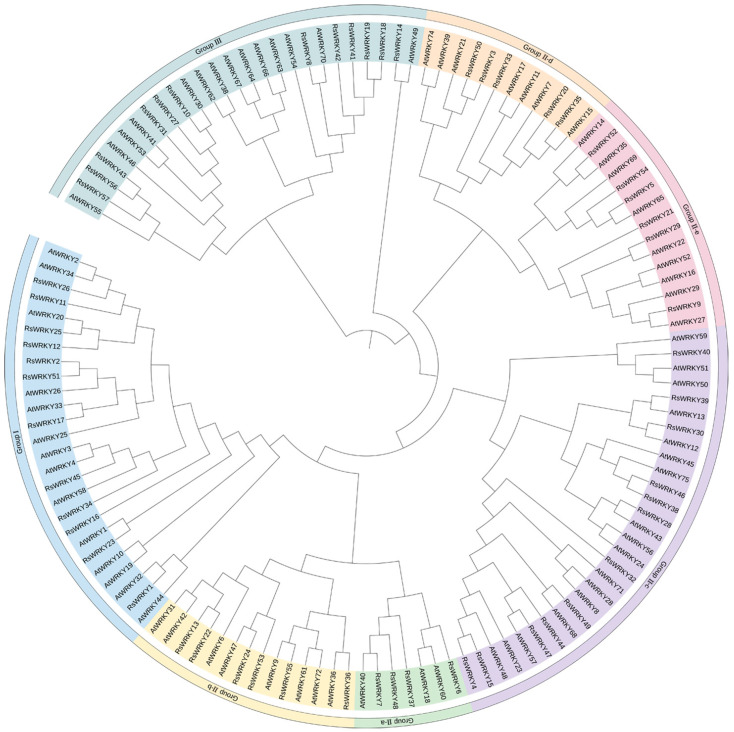
Phylogenetic analysis among the identified WRKY-conserved proteins in *A. thaliana* and *R. simsii*. The 72 *A. thaliana* and 57 *R. simsii* WRKY sequences were aligned using Muscle; the phylogenetic tree was constructed by MEGA6.0 with the maximum likelihood method, and the bootstrap value was set at 1000 repetitions. The colorful regions represent different subfamilies.

**Figure 3:**
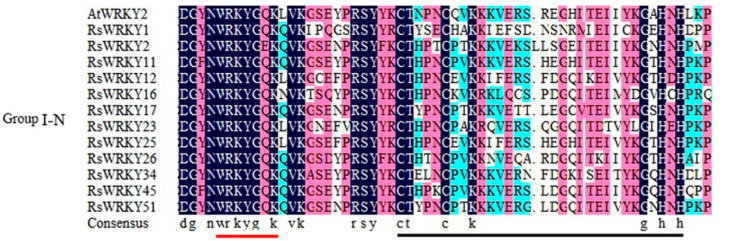

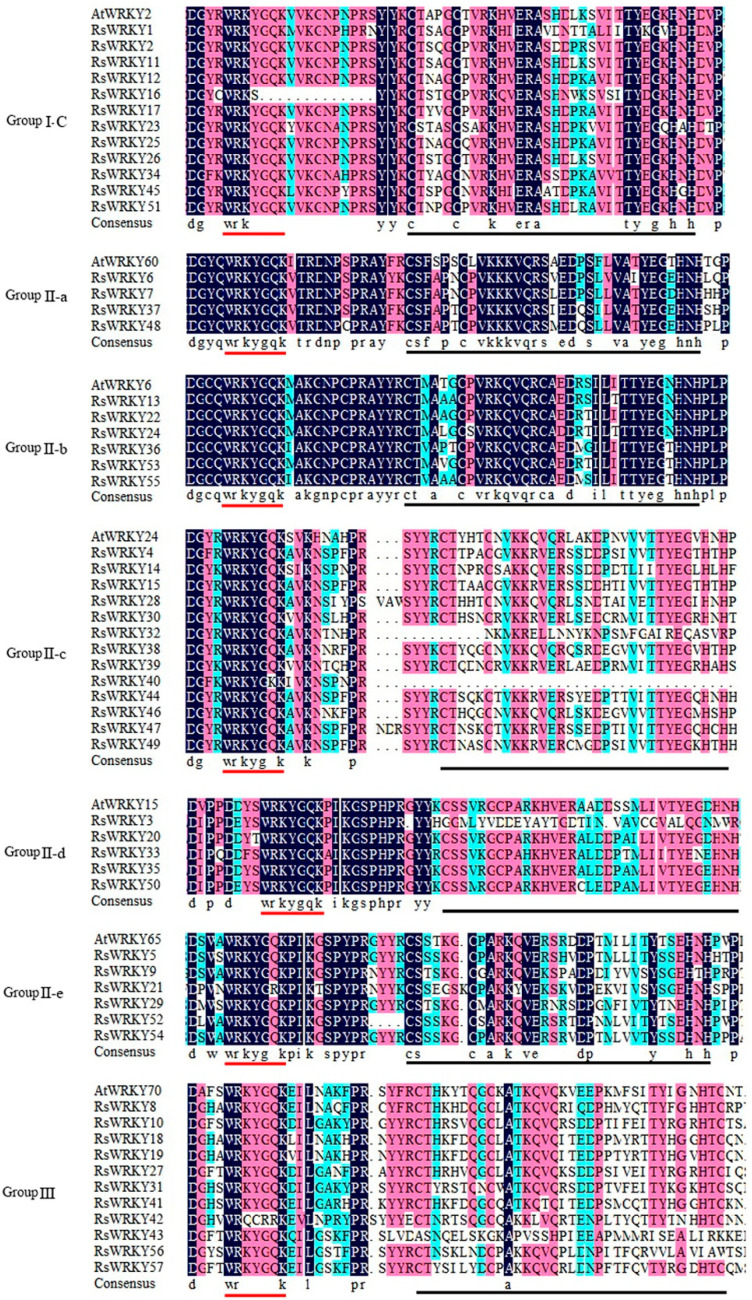
Multiple alignments of RsWRKY proteins and selected AtWRKY amino acid sequences. “-N” and “-C” indicate the N-terminal and C-terminal WRKY domains of Group I WRKY members, respectively. The red solid line represents the highly conserved WRKYGQK heptapeptide, and the black solid line represents the zinc finger domain.

To further analyze the evolutionary relationship of the RsWRKY family, 57 RsWRKY proteins and 72 AtWRKY proteins were examined by MEGA6.0 software (Figure 2). Seven AtWRKYs were selected as representatives of *A. thaliana* and made multiple sequence alignments with RsWRKYs (Figure 3). The RsWRKYs proteins were classified into three main groups based on the group features of the WRKY superfamily in *Arabidopsis* [3]. With the exception of RsWRKY16, which originated from Group I but lost one WRKY domain, the 12 RsWRKYs that comprised two WRKY domains and a C2H2 zinc finger motif (C-X4-C-X22-23-HXH) were assigned into Group I, an N-terminal WRKY domain (NTWD), and a C-terminal WRKY domain (CTWD). The same situation occurred in *Arabidopsis* as well [29]: the deletion of the WRKY domain in the N-terminal is found in *Arabidopsis*, but the deletion in the C-terminal is found in the RsWRKY family. Group II is the largest group, which is classified into five subgroups with a single WRKY domain and a zinc finger motif. A total of 4, 6, 13, 5, and 6 RsWRKYs clustered in II-a, II-b, II-c, II-d, and II-e, respectively. Among them, II-a and II-b clustered in one clade, while subgroups II-d and II-e clustered in another clade. RsWRKY14 is a member of Group II-c but is more similar to Group III from the same branch based on the phylogenetic analysis and so clustered into II-c when multiple sequences were aligned. The WRKY domain (WRKYGQK, Motif 1) was highly conserved among the 55 proteins, and only two of them contained variations. Thirty-four RsWRKYs harbored the motif 1 sequence, while RsWRKY40 and RsWRKY21 had the WRKYGKK and WRKYGRK sequences instead. The remaining 11 RsWRKYs were distributed into Group III.

### 2.4. Gene Structure and Motif Composition of RsWRKY Gene Family

**Figure 4:**
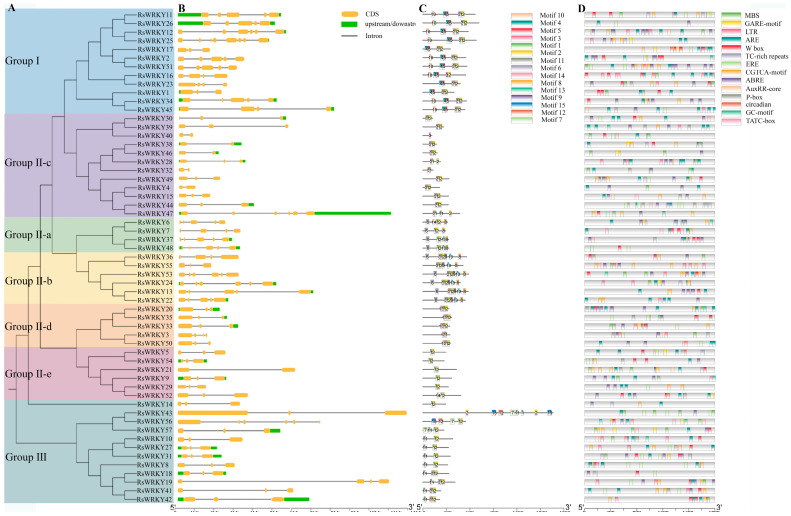
Phylogenetic tree, gene structure, distribution of conserved motif, and cis-regulatory elements of RsWRKY gene family. (**A**) Phylogenetic tree was constructed by MEGA6.0 software and shown on the left. (**B**) Schematic of gene structure was displayed by the gene structure display server, CDSs, UTRs, and introns are represented by orange boxes, green boxes, and black lines. (**C**) The conserved motifs of RsWRKY proteins and different motifs are represented by different color boxes, and numbers. (**D**) Cis-acting element analysis of promoter region of RsWRKY gene family.

MEGA6.0 was used to construct an evolutionary tree (Figure 4A), the result was consistent with the evolution of *Arabidopsis thaliana* (Figure 2), which revealed that WRKY proteins were highly conserved in evolution. *RsWRKY* family members are mainly distributed in several different branches, which have differences and similarities in evolution. All *RsWRKY* genes possessed one to seven introns. Overall, 26 (45.6%) genes contained two introns, 24 (42%) had three to six introns, while seven introns were found in *RsWRKY25* (Figure 4B). 

To examine the number and types of motifs contained in RsWRKY proteins and their homologous protein pairs in different groups, 57 WRKY protein members were analyzed by the online software MEME, and the maximum number of motifs was set at 15. The length of 15 motifs ranged roughly between 21 and 50 amino acids, as shown in (Figure S1). The heptapeptide stretch WRKYGQK, which was considered as an essential characteristic of the WRKY family, was present in motif 1 and motif 4. Proteins in the same group had similar numbers and types of motifs. Except for the RsWRKY16, motifs 1, 2, 3, 4, and 5 were found in Group I. Motif 6 was identified as nuclear localization signals (NLS), which was observed in II-a and II-b; meanwhile, motif 8 was unique to these two groups, and motif 11 was only present in Group III (Figure 4C). 

Cis-acting elements on promoters are functional elements that regulate gene expression. We downloaded the 2 kb promoter region sequence upstream of the initiation codons of the *RsWRKY* genes and used PlantCARE to identify the cis-regulatory elements in there (Figure 4D). Interestingly, in addition to a large number of basic TATA-box, CAAT-box core elements, and light-response elements, there are numerous hormones, stress response, and regulation of growth and development elements. Elements that are involved in defense and stress response include TC-rich repeats, the MYB binding site involved in drought induction (MBS), low-temperature-response element (LTR), essential regulator element for anaerobic induction (ARE), and hypoxia-specific induction-related element (GC-motif). Other types of cis-elements, such as auxin-reaction element (AUXRR-core), abscisic-acid-reaction element (ABRE), estrogen-response element (ERE), gibberellin-reaction element (TATC-box, P-box, and GARE-motif), and methyl-jasmonate-reaction elements (CGTCA-motif), are hormone-related elements. Cis-acting element analysis indicated that WRKY family genes of *R. simsii* may be closely related to biological stress, abiotic stress response, hormone induction, and plant growth and development.

### 2.5. Expression Profile of RsWRKY Genes under GA and MeJA Treatment 

A phylogenetic tree was constructed between the RsWRKY family and the AtWRKY family in our studies to screen candidate genes associate with abiotic stress for subsequent analysis (Figure 2). For qRT-PCR, we selected the *RsWRKY* genes that clustered with the reported *AtWRKY* resistance-related genes. Following cis-acting element analysis, it was discovered that some *RsWRKYs* contain gibberellin-reaction elements (TATC-box, P-box, and GARE-motif) as well as methyl-jasmonate-reaction elements (CGTCA-motif). To investigate the relationship between *RsWRKYs* and phytohormone-signaling molecules, the expression levels of *RsWRKYs* were analyzed by qRT-PCR under GA and MeJA treatment. 

**Figure 5:**
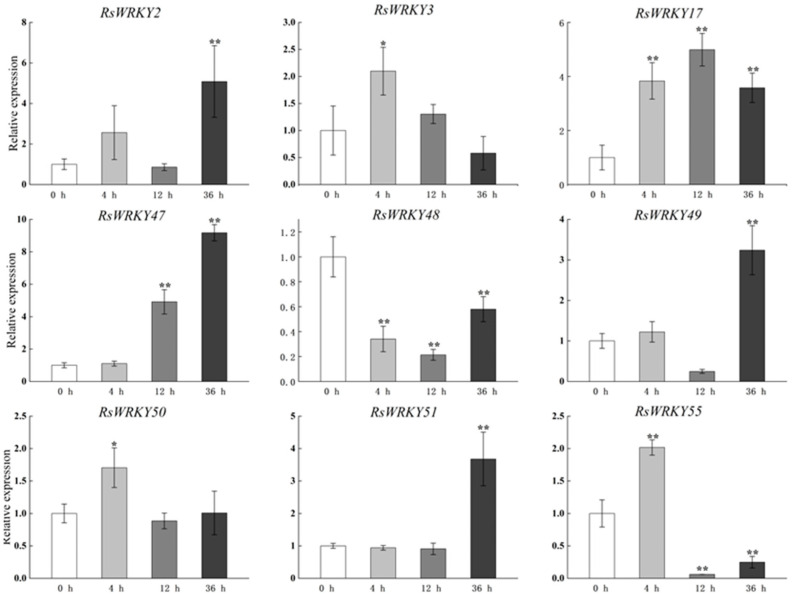
Expression profiles of *RsWRKY* genes under 100 µM GA treatment. Gene expression at 0 h was normalized as “1.” Data are mean ± standard deviation (SD), calculated from three biological replicates, and vertical lines represent the standard deviation. * and ** mean significant difference at *p* < 0.05 and *p* < 0.01, respectively.

Other genes were expressed after GA treatment, as shown in Figure 5, with the exception of *RsWRKY48*, which was down-regulated. Obviously, the expression levels of *RsWRKY2*, *RsWRKY47*, *RsWRKY49*, and *RsWRKY51* reached the highest value at 36 h after GA hormone treatment, which was much higher than that of the control. *RsWRKY3*, *RsWRKY50*, and *RsWRKY55* reached their highest point after 4 h GA treatment. However, *RsWRKY17* was significantly expressed throughout the treatment period.

**Figure 6:**
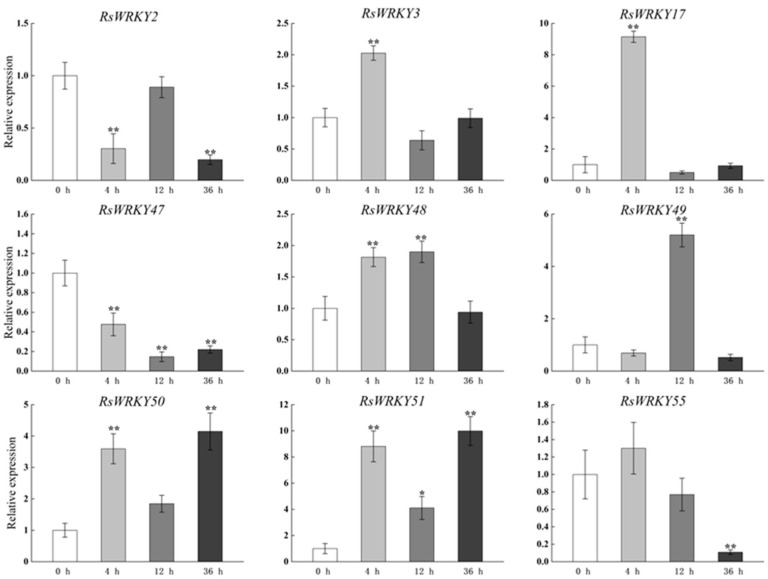
Expression profiles of *RsWRKY* genes under 200 µM MeJA treatment. Gene expression at 0 h was normalized as “1.” Data are mean ± standard deviation (SD), calculated from three biological replicates, and vertical lines represent the standard deviation. * and ** mean significant difference at *p* < 0.05 and *p* < 0.01, respectively.

*RsWRKY3*, *RsWRKY17*, *RsWRKY48*, *RsWRKY50*, and *RsWRKY51* all responded to MeJA treatment with similar expression patterns, and their expression levels were all up-regulated after 4 h treatment. However, after MeJA treatment, *RsWRKY2*, *RsWRKY47*, and *RsWRKY55* showed an obvious decreasing trend (Figure 6). 

### 2.6. Expression Profile of RsWRKY Genes under Heat and Drought Treatment

Heat and drought are increasingly limiting factors for plant growth and development as a result of global warming. Therefore, it is urgent to explore *Rhododendron* stress-resistance genes for *Rhododendron* breeding and application.

**Figure 7:**
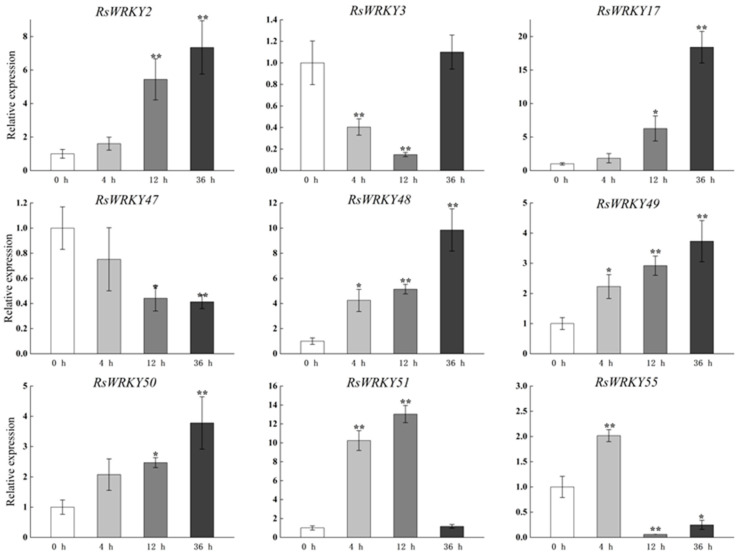
Expression profiles of *RsWRKY* genes under heat treatment. Gene expression at 0 h was normalized as “1.” Data are mean ± standard deviation (SD), calculated from three biological replicates, and vertical lines represent the standard deviation. * and ** mean significant difference at *p* < 0.05 and *p* < 0.01, respectively.

**Figure 8:**
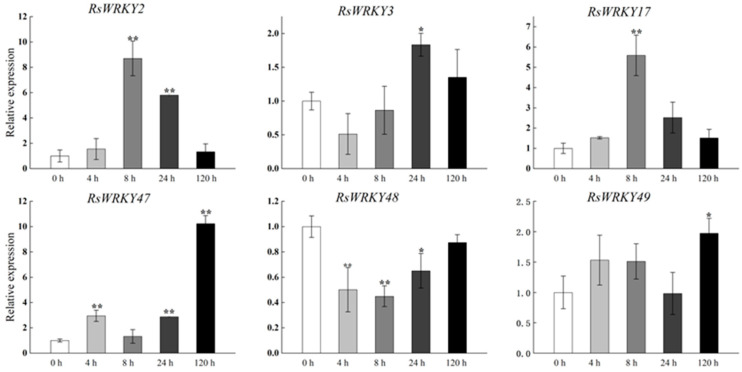

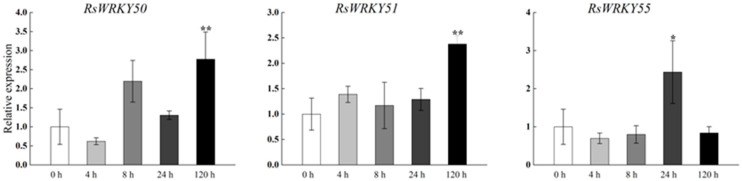
Expression profiles of *RsWRKY* genes under drought treatment. Gene expression at 0 h was normalized as “1.” Data are meant ± standard deviation (SD), calculated from three biological replicates, and vertical lines represent the standard deviation. * and ** mean significant difference at *p* < 0.05 and *p* < 0.01, respectively.

Under high-temperature treatment, *RsWRKY2*, *RsWRKY17*, *RsWRKY48*, *RsWRKY49*, and *RsWRKY50* were up-regulated at all five time points. *RsWRKY51* and *RsWRKY55* up-regulated first and then dropped rapidly, while *RsWRKY3* showed an opposite response to high temperature (Figure 7). At the same time, the drought treatment of *Rhododendrons* was carried out by withholding the irrigation. *RsWRKY2* and *RsWRKY17* showed noticeable up-regulation at 8 h, and the expression level of *RsWRKY2* was over seven-fold higher at 8 h drought treatment. *RsWRKY47*, *RsWRKY49*, *RsWRKY50*, and *RsWRKY51* genes were mainly induced on the fifth day of drought treatment; notably, *RsWRKY3* and *RsWRKY55* first showed a downward trend at the initial stage but began to reach the highest value on the first day of drought (Figure 8).

In summary, we identified some *RsWRKY* genes that may potentially play a vital role in heat- and drought-stress resistance. This study can provide a meaningful reference for *Rhododendron* breeding improvement.

## 3. Discussion

Transcription factors, also known as trans-acting factors [29], exist in the form of a gene superfamily and play a critical regulatory role in the growth and development of plants and their response to the external environment. Thus far, the research on the identification and analysis of transcription factor families from the whole genome is increasing, which has become one of the focuses of genomics research [30], aiming to provide a reference for further study. The WRKY family is one of the ten transcription factor families in higher plants, and WRKY is famous for its highly conserved sequence (WRKYGQK), which is essential for WRKY transcription factor recognition and binding to the W-box element at the target gene promoter [3,7,31]. WRKY transcription factors are involved in a wide range of plant biological processes, including plant growth and development, stress-response mechanism, and secondary metabolite synthesis [32]. With the publication and improvement of plant genome data, many plant WRKY gene family members have been identified at the genome-wide level, and WRKY families were identified in *Arabidopsis* (72), tomato (104), cucumber (61), *Populus trichocarpa* (122), and rice (102) [1,2,33,34,35]. 

In this study, 57 *WRKY* genes (*RsWRKY1–RsWRKY57*) were identified in the genome data of *R. simsii* (Table S1). The phylogenetic tree was constructed with 57 *RsWRKY* genes and 72 *AtWRKY* genes, and genes with similar structures were clustered into Groups I, II, and III, respectively (Figure 2). Thirty-four members of Group II were divided into five subgroups: II-a, II-b, II-c, II-d, and II-e. This is consistent with the grouping of model plants *Arabidopsis thaliana* [10]. In the conserved heptapeptide structure, RsWRKYs had three types of variations (Figure 3). These variations may cause the protein to fail to bind to downstream genes or even lose their gene function. For example, in soybean, GmWRKY6 and GmWRKY21 fail to bind to the downstream gene W-box due to structural changes in the heptapeptide [36]. In terms of protein quantity, proteins of II-c were the largest, accounting for 22.8% of all proteins, followed by proteins of I. Except for *RsWRKY16*, other members of the Group I all had two WRKY-conserved domains, suggesting that *RsWRKY16* may have lost one WRKY-conserved domain during evolution. The loss of the WRKY domain also occurred in the evolution of *Arabidopsis*, as *AtWRKY10* in Group I only had one WRKY domain [14]. Group I is the original ancestor of Group II and Group III in the evolution of the plant WRKY gene family, which are mainly found in lower plants [5]. The gene function of Group I was more conserved than that of Group II and III [37,38]. In terms of evolution, *RsWRKYs* of Group II are more active than *AtWRKYs* of *Arabidopsis thaliana* Group II, which is the main driving force of the expansion of the RsWRKY gene family. It is speculated that members of this group may play specific functions in *Rhododendron*. Chromosomal mapping analysis showed that *RsWRKYs* were found on the 13 chromosomes except for chromosome 9 (Figure 1), and this similar phenomenon was also found in grape [39]. It is supposed that the sequencing accuracy and assembly of the genome are not perfect in *R. simsii*, *RsWRKY55–RsWRKY57* sequences cannot be located, and they may be distributed on any chromosome.

A conserved domain motif 1 was discovered in all members of the RsWRKY family through the results of conserved motif analysis and gene structure analysis, which is likely to be the core conserved domain of the RsWRKY family (Figure 4C). Previous studies suggested that the gene encoding one WRKY domain evolved from the gene encoding two WRKY domains; the WRKY domain at the C-terminal is highly conserved, which plays a leading role in determining the binding process of DNA [40]. This explains why the number of proteins containing two WRKY domains is much smaller than that containing one WRKY domain. Cis-acting elements are important molecular switches that regulate gene expression. Comprehensive analysis of the type and number of cis-acting elements can better elucidate the expression patterns and potential biological functions of genes [41,42,43]. We predicted cis-acting elements in these *RsWRKY* genes and found a variety of cis-elements (Figure 4D), including light-response element, hormone-response element, and stress-response element. We speculate that optical signals, hormones, and biotic and abiotic stress may activate *RsWRKY* genes for expression, which directly or indirectly regulates various biological processes. Numerous studies have demonstrated that *WRKYs* can increase resistance to biotic and abiotic stresses, such as osmotic, drought, salt, cold, heat, and UV-B stress [44,45,46,47,48,49]. To further identify candidate genes involved in hormone signaling pathways or abiotic stress responses, we used the qRT-PCR method to measure their expression levels in response to the hormone, drought, and high-temperature treatments. Hormones play a crucial role in plant growth and development and abiotic stress responses. For example, *AtWRKY11*, *AtWRKY17*, and *AtWRKY70* are essential components of the antagonistic action between JA and SA [25,50]. *OsWRKY3* and *OsWRKY12* are up-regulated in response to JA, SA, benzothiadiazole, and so on [51]. Furthermore, overexpression of *AtWRKY39* in *Arabidopsis* can improve its tolerance to heat stress, and expression of *AtWRKY39* is induced by SA and MeJA and enhances plant heat tolerance [48]. In *Camellia sinensis*, *CsWRKY2* is involved in ABA downstream signaling pathways and drought tolerance [52]. In this study, *RsWRKYs* responded to exogenous GA and MeJA, and nine *RsWRKYs* can respond to one of them. *RsWRKY49* and *RsWRKY51* exhibited a similar trend under GA treatment; moreover, *RsWRKY50* and *RsWRKY51* were also mainly expressed at 4 h and 12 h treatment (Figure 5 and Figure 6). There are many abiotic stresses that can cause osmotic stress in plants, such as drought, high-salt, and high-temperature stress. The specific expression of a gene usually reflects its corresponding function. In *Vitis vinifera*, the expressions of *VvWRKY24* and *VvWRKY49* increased under drought stress, while other genes did not change significantly [53]. Therefore, high temperature and drought were treated as abiotic stresses in this study. *RsWRKY17*, *RsWRKY49*, *RsWRKY50*, and *RsWRKY51* were expressed under all treatments; *RsWRKY17* was the most responsive to high-temperature treatment (Figure 7). *RsWRKY2* and *RsWRKY17* were significantly up-regulation at 8 h of drought stress, while *RsWRKY47*, *RsWRKY49*, *RsWRKY50*, and *RsWRKY51* genes were mainly expressed on the fifth days (Figure 8). qRT-PCR results showed that differential expression of some *RsWRKY* genes under hormone and abiotic stress treatments highlight the extensive involvement of *WRKY* genes in environmental adaptation. We speculated that *RsWRKYs* may enhance its resistance to drought and heat stress. Whether a single ***R****sWRKY* acts alone or multiple *RsWRKYs* act together under drought stress needs to be further studied. The above findings suggest that transcription factors can act through their involvement in hormone synthesis or hormone-signaling networks. In summary, our findings provide a valuable resource for select candidate *RsWRKY* genes, which can facilitate the further functional studies of *RsWRKYs* involved in various biological stresses. Currently, since genetic transformation systems have not been established in most plants, it is necessary to explore the function of genes in enhancing plant resistance by performing functional validation in model plants and analyzing the effects of genes on plant phenotype and physiology [54,55]. Interestingly, the specific regulatory mechanisms in *Rhododendron* needs to be further studied.

## 4. Materials and Methods

### 4.1. Plant Materials and Growth Condition

The *Rhododendron* “FengGuan” was used throughout the study, which was planted in an artificial climate incubator with a 14 h light/10 h dark photoperiod, 600 μmol m^−2^ s^−1^ light intensity, 25 ℃ day/18 ℃ night, and 75% humidity at the JIYANG College of Zhejiang A&F University, Zhejiang, China (29°44′51″ N, 120°15′17″ E). Plants of uniform size were subjected to the hormone, drought, and heat stresses in this experiment. Gibberellic acid (Sigma, Darmstadt, Germany) was dissolved in 1 ml ethanol, then diluted with deionized water, obtaining a final solution with ethanol:water(*v*/*v*) ratio of 1:2000 in a solution containing 0.02% Tween-20. Methyl jasmonate (Aladdin, Shanghai, China) solution was prepared in the same way as gibberellin. Hormone treatments were carried out by spraying leaves with 100 µM GA and 200 µM MeJA, respectively; leaves were sampled at 0 h, 4 h, 8 h, 12 h, and 36 h. For drought treatment, irrigation was stopped for 5 days and sampled after 0 h, 4 h, 12 h, 24 h, and 120 h of treatment. For temperature stress, plantlets were exposed to 43 °C and sampled as above for hormone treatment. The collected leaf samples were frozen in liquid nitrogen and stored at −80 °C.

### 4.2. Identification and Physicochemical Properties of WRKY Genes from R. simsii

Putative *RsWRKY* gene sequences were downloaded from *R. simsii* genome data [56], and the *Rhododendron* genome database (http://bioinfor.kib.ac.cn/RPGD/index.html, accessed on 10 June 2022) has been comprehensively described [57]. All sequences were sent to NCBI (https://www.ncbi.nlm.nih.gov/Structure/cdd/wrpsb.cgi, accessed on 10 June 2022) [58] to ensure whether they contained a WRKY-conservative domain; conservative domain prediction software PFAM (http://pfam.xfam.org/, accessed on 10 June 2022) [59] and SMART (http://smart.embl-heidelberg.de/, accessed on 10 June 2022) [60] were used to eliminate duplicate and incomplete sequences. We used PlantRegMap to identify WRKY family members in the *R. simsii* genome and obtained 63 potential genes that were in accord with the genes listed in the supplementary table by Yang [56]. Four genes (*Rhsim10G0148700*, *Rhsim07G0087600*, *Rhsim13G0004700*, and *Rhsim11G0062400*) were discovered to lack the WRKYGQK-conserved domain following motif analysis of these 63 putative genes utilizing multiple sequence alignment and online software MEME. Therefore, we removed the aforementioned genes. Additionally, we discovered that *Rhsim10G0148800* and *RsWRKY40 (Rhsim10G0148600)* share a 91.3% similarity in their nucleotide and amino acid sequences. Analogously, *RsWRKY55 (RhsimUnG0056300)* and *RhsimUnG0199600* are comparable, with the comparison similarity reaching 93.12%. The definition of tandem repeat genes by Huang [2] stated that a gene is classified as tandem repeat if the physical distance between two or more homologous genes on the chromosome is less than 100 kb. In view of the similarity and the chromosome, we manually deleted these two repetitive sequences. The physicochemical properties of *RsWRKYs* members were analyzed by the ProtParam tool on ExPASY (https://web.expasy.org/protparam/, accessed on 10 June 2022) [61]. Including the number of amino acids, molecular weight, theoretical isoelectric point, aliphatic index, and grand average of hydropathicity, the subcellular localization of RsWRKYs was predicted by WoLF PSORT (https://wolfpsort.hgc.jp/, accessed on 10 June 2022) and PLOC (http://www.csbio.sjtu.edu.cn/bioinf/Cell-PLoc, accessed on 10 June 2022). 

### 4.3. Chromosomal Location of RsWRKY Genes in R. simsii

After obtaining the mapping information of the RsWRKY gene family on chromosomes from the DNA annotation file of the *R. simsii* genome database [57], the online tool MG2C (http://mg2c.iask.in/mg2c_v2.0/, accessed on 10 June 2022) was used to visualize the distribution of *RsWRKY* genes on barely 13 chromosomes.

### 4.4. Multiple Sequence Alignment and Phylogenetic Analysis of RsWRKYs

The WRKY proteins of *Arabidopsis thaliana* were retrieved from TAIR (http://www.arabidopsis.org/, accessed on 10 June 2022). Multiple sequence alignments between RsWRKYs and AtWRKYs were first performed using DNAMAN, and the MEGA6 software was used to construct a phylogenetic tree using the maximum likelihood (ML) method [62], with partial deletion of 1000 bootstraps and a WAG model. iTOL (https://itol.embl.de/, accessed on 10 June 2022) [63] was used to visualize and beautify the phylogenetic tree. 

### 4.5. Analysis of the Gene Structure, Conserved Motifs, and Cis-Acting Elements of RsWRKYs

The website Gene Structure Display Server (GSDS) (http://gsds.cbi.pku.edu.cn/, accessed on 10 June 2022) was employed to determine the exon-intron distribution from DNA sequences and the coding domain sequences (CDS). A common motif in a gene family is likely to be a key sequence that performs the corresponding function or an essential sequence in the gene family. The conserved motifs of 57 *RsWRKYs* were detected and analyzed by MEME (http://meme-suite.org/tools/meme, accessed on 10 June 2022) [64]. The number of motif software parameters was set to 15, and the default values for other parameters were used. Fifty-seven promoter sequences corresponding to WRKY upstream 2 kb were extracted from *R. simsii* chromosome genome data; then, we used PlantCARE (https://bioinformatics.psb.ugent.be/webtools/plantcare/html/, accessed on 10 June 2022) [65] to identify transcription factor binding sites. To analyze promoter specificity under multiple abiotic stresses, low temperature, heat stress, drought, hormones responses, and salt response, motifs were selected. 

### 4.6. Expression Profile Analysis of RsWRKY Genes by qRT-PCR 

Total RNA was isolated by FastPure Plant Total RNA Isolation Kit (Polysaccharides and Polyphenolics-rich) (Vazyme, Nanjing, China); then, reverse 1 μg DNA-free RNA was transcribed into cDNA using HiScript III All-in-one RT SuperMix Perfect for qPCR (Vazyme). Gene-specific primers (Table S2) were designed using Primer Premier5 and synthesized by Tsingke (Beijing, China). Individual qRT-PCR reactions contained 5 μL 2 × ChamQ Universal SYBR qPCR Master Mix, 2 μL cDNA of 25-times diluted cDNA, and 0.2 μL forward and reverse primers and was filled with deionized water to 10 μL. The reaction was carried out as follows: 30 s at 95 °C for denaturation, followed by 40 cycles of 10 s at 95 °C, 30 s at 60 °C, and 30 s at 72 °C. The glyceraldehyde 3-phosphate dehydrogenase (*GAPDH*) gene was used as an internal control [66] to normalize the measured gene expression levels based on the standard curves, and the Ct values were automatically calculated by LightCycler^®^ 480 II (Roche Diagnostics, Germany) software. Finally, the relative expression of the target gene was calculated according to the 2^−ΔΔCT^ method [67]. SPSS 20.0 software (IBM Corporation, Armonk, NY, USA) was used to perform one-way ANOVA for all of the experimental data, and Duncan’s test was used to count the significant difference among all samples (*p* < 0.05). Then, all data were visualized via Origin 9.0 (Northampton, MA, USA). Three biological replicates were performed for each sample to ensure reliability.

## 5. Conclusions

In this paper, we identified 57 RsWRKY transcription factors at the genome level. Physicochemical properties, gene structure, motif composition, cis-acting elements, chromosomal localization, phylogenetic relationship, and differential gene expression under four abiotic stresses were demonstrated in this study. Phylogenetic tree analysis showed that RsWRKY family members were divided into seven groups (I, II a–e, and III) with similar motif-distribution patterns. Promoter analysis revealed that RsWRKYs contained cis-regulatory elements related to hormones and stress response. RsWRKYs were highly conserved in the evolutionary process, and most of them were distributed on 13 chromosomes of *R. simsii*. We found that *RsWRKYs* in this family were highly conserved at gene structure and evolutional levels. Expression profiles of *RsWRKY* genes responding to hormone and abiotic stresses were observed. Our results may contribute to selecting appropriate candidate genes involved in hormones and stress responses in *Rhododendron* species and to lay a foundation for mechanism research and practical application of the WRKY transcription factor in *Rhododendron* through the combined efforts of our group.

## Data Availability

The data presented in this study are available on request from the corresponding author. The data are not publicly available due to privacy.

## Supplementary Materials

The following supporting information can be downloaded at: https://www.mdpi.com/article/10.3390/plants11212967/s1, Figure S1: The specific sequence information of 15 motifs. The letters represent amino acids, the color does not represent any meaning, which are automatically synthesized by the MEME, and the height of the letters represents the motifs conservation, Table S1: Physicochemical properties of WRKY transcription factors in *Rhododendron simsii*, Table S2: Primers of the RsWRKY genes for qRT-PCR.
