# Supplementary material for: Comprehensive Genomic Survey, Structural Classification, and Expression Analysis of WRKY Transcription Factor Family in Rhododendron simsii
Source: Plants (Basel). 2022 Nov 3;11(21):2967. doi: 10.3390/plants11212967 (PMC9654210; doi:10.3390/plants11212967)
Supplement: Supplementary file 1 [file plants-11-02967-s001.zip › plants-1949281-supplementary.pdf]

Table S1. Physicochemical properties of WRKY transcription factors in *Rhododendron simsii.*

Gene ID	Gene name	Length	MW	pI	AI	GRAVY	Subcellular localization
Rhsim01G0033600	RsWRKY1	397	43507.84	9.07	61.64	-0.763	Nucleus
Rhsim01G0241100	RsWRKY2	548	60491.94	8.44	43.27	-0.965	Nucleus
Rhsim01G0268400	RsWRKY3	346	38661.99	9.75	70.98	-0.703	Nucleus
Rhsim02G0002700	RsWRKY4	213	24291.62	9.44	49.39	-0.559	Nucleus
Rhsim02G0146200	RsWRKY5	293	32787.62	5.71	56.86	-0.829	Nucleus
Rhsim02G0213700	RsWRKY6	316	34943.16	5.57	65.70	-0.658	Nucleus
Rhsim02G0213800	RsWRKY7	291	31898.76	7.57	70.07	-0.615	Nucleus
Rhsim03G0153300	RsWRKY8	315	34659.50	5.61	61.24	-0.649	Nucleus
Rhsim03G0192300	RsWRKY9	365	39330.27	4.94	58.85	-0.673	Nucleus
Rhsim03G0198800	RsWRKY10	382	41873.23	5.96	55.45	-0.757	Nucleus
Rhsim04G0007000	RsWRKY11	669	72732.2	5.8	53.41	-0.786	Nucleus
Rhsim04G0015000	RsWRKY12	579	62823.53	6.61	54.39	-0.709	Nucleus
Rhsim04G0052400	RsWRKY13	578	62485.31	6.54	62.23	-0.751	Nucleus
Rhsim04G0158500	RsWRKY14	292	32519.94	5.18	60.79	-0.796	Nucleus
Rhsim04G0240600	RsWRKY15	328	36685.79	6.19	46.62	-0.890	Nucleus
Rhsim05G0168200	RsWRKY16	545	59270.62	8.45	56.95	-0.743	Nucleus
Rhsim05G0226500	RsWRKY17	351	39397.12	8.62	39.74	-1.162	Nucleus
Rhsim05G0231400	RsWRKY18	330	36624.38	5.93	62.88	-0.735	Nucleus
Rhsim05G0231500	RsWRKY19	410	45369.44	6.41	63.24	-0.687	Nucleus
Rhsim06G0054700	RsWRKY20	331	36187.15	9.73	67.49	-0.539	Nucleus
Rhsim06G0156700	RsWRKY21	429	46482.07	8.36	56.34	-0.708	Nucleus
Rhsim06G0164400	RsWRKY22	540	58808.43	6.52	59.57	-0.684	Nucleus
Rhsim06G0174100	RsWRKY23	476	52130.14	6.61	54.89	-1.038	Nucleus
Rhsim06G0220000	RsWRKY24	478	52563.86	8.03	63.7	-0.656	Nucleus
Rhsim06G0226600	RsWRKY25	681	74141.16	6.31	65.87	-0.638	Nucleus
Rhsim06G0235700	RsWRKY26	716	77801.64	6.04	58.59	-0.775	Nucleus
Rhsim07G0005900	RsWRKY27	327	36699.90	5.34	59.36	-0.603	Nucleus
Rhsim07G0009800	RsWRKY28	223	25194.37	7.69	62.11	-0.770	Nucleus
Rhsim07G0010600	RsWRKY29	325	36112.15	5.61	53.14	-0.821	Nucleus
Rhsim07G0037500	RsWRKY30	125	14916.85	9.61	45.84	-1.154	Nucleus
Rhsim07G0083100	RsWRKY31	351	39662.88	5.89	57.49	-0.827	Nucleus
Rhsim07G0087300	RsWRKY32	129	14325.20	10.12	57.44	-0.858	Nucleus
Rhsim07G0225700	RsWRKY33	343	37245.96	9.25	65.07	-0.535	Nucleus
Rhsim08G0072700	RsWRKY34	557	61388.34	6.59	61.42	-0.733	Nucleus
Rhsim08G0098300	RsWRKY35	368	40581.11	9.36	71.30	-0.468	Nucleus
Rhsim08G0158800	RsWRKY36	559	60642.98	5.94	61.48	-0.721	Nucleus
Rhsim08G0205900	RsWRKY37	330	36437.58	8.44	64.70	-0.795	Nucleus
Rhsim10G0005100	RsWRKY38	176	20136.78	9.58	57.50	-0.812	Nucleus
Rhsim10G0147700	RsWRKY39	264	29901.62	8.68	58.37	-0.690	Nucleus
Rhsim10G0148600	RsWRKY40	115	12866.11	5.45	50.09	-0.972	Nucleus
Rhsim11G0062500	RsWRKY41	227	25450.47	6.75	66.65	-0.671	Nucleus
Rhsim11G0064200	RsWRKY42	214	24134.05	9.59	65.19	-0.857	Nucleus
Rhsim11G0120500	RsWRKY43	1661	190287.09	5.92	88.49	-0.318	Nucleus
Rhsim12G0007700	RsWRKY44	325	36659.89	6.94	41.72	-1.149	Nucleus
Rhsim12G0077700	RsWRKY45	535	57646.36	6.61	53.83	-0.813	Nucleus
Rhsim12G0094500	RsWRKY46	184	20827.28	9.52	50.82	-0.877	Nucleus
Rhsim12G0134800	RsWRKY47	471	51888.61	8.77	55.97	-0.739	Nucleus
Rhsim12G0187400	RsWRKY48	331	36676.08	8.69	63.63	-0.759	Nucleus

Rhsim13G0003300	RsWRKY49	333	37017.52	6.60	61.23	-0.648	Nucleus
Rhsim13G0061600	RsWRKY50	353	39625.09	9.76	66.54	-0.747	Nucleus
Rhsim13G0063200	RsWRKY51	562	61647.22	7.66	47.21	-0.932	Nucleus
Rhsim13G0125600	RsWRKY52	484	52564.64	5.28	51.61	-0.892	Nucleus
Rhsim13G0151900	RsWRKY53	583	62994.81	6.58	59.18	-0.668	Nucleus
Rhsim13G0187900	RsWRKY54	271	29959.99	5.45	49.63	-0.820	Nucleus
RhsimUnG0056300	RsWRKY55	513	55840.93	6.62	63.22	-0.668	Nucleus
RhsimUnG0080500	RsWRKY56	547	63550.07	5.59	94.42	-0.290	Nucleus
RhsimUnG0083000	RsWRKY57	268	30899.98	5.33	54.59	-0.759	Nucleus

Table S2. Primers of the *RsWRKY* genes for qRT-PCR

Gene	Primer name	Primer sequences (5′-3′)
RsWRKY2	RsWRKY2-qF	GAGGGAACCAAGAGTTGTAGTC
	RsWRKY2-qR	GACGACCTTCTGCCCATATT
RsWRKY3	RsWRKY3-qF	GAGTGTAGCCTGAGAGTTGTAAG
	RsWRKY3-qR	CCTCTCCTGTTTCCACCATTAT
RsWRKY17	RsWRKY17-qF	ATGCACGTACCCGAATTGT
	RsWRKY17-qR	TGGTTGTGGCTGCCTTTAT
RsWRKY47	RsWRKY47-qF	CCGAAGATCCCACCATAGTAATC
	RsWRKY47-qR	AGCAGTGATCAGTCCAACAC
RsWRKY48	RsWRKY48-qF	AACCCTTGTCCAAGAGCTTAC
	RsWRKY48-qR	GTGGTTGTGCTCTCCTTCAT
RsWRKY49	RsWRKY49-qF	TGTGAAGAAGAGAGTGGAGAGA
	RsWRKY49-qR	AGTTGGCTGTGATGGGTATG
RsWRKY50	RsWRKY50-qF	GGCTCTTCTGACCAGATTTCTC
	RsWRKY50-qR	CGACCACTACTCCCACATTTC
RsWRKY51	RsWRKY51-qF	CAGCGGTAACCACTCCATAAA
	RsWRKY51-qR	GAAGACTGGTGGTGGAACATAG
RsWRKY55	RsWRKY55-qF	GAATCAGCGAAAGCCGAAATG
	RsWRKY55-qR	GCATATGGAGGGATCGGTAATC
GAPDH	GAPDH-qF	GTTCTGCCAGCGCTTAATGG
	GAPDH-qR	GCCTTCTCAAGCCTCACAGT

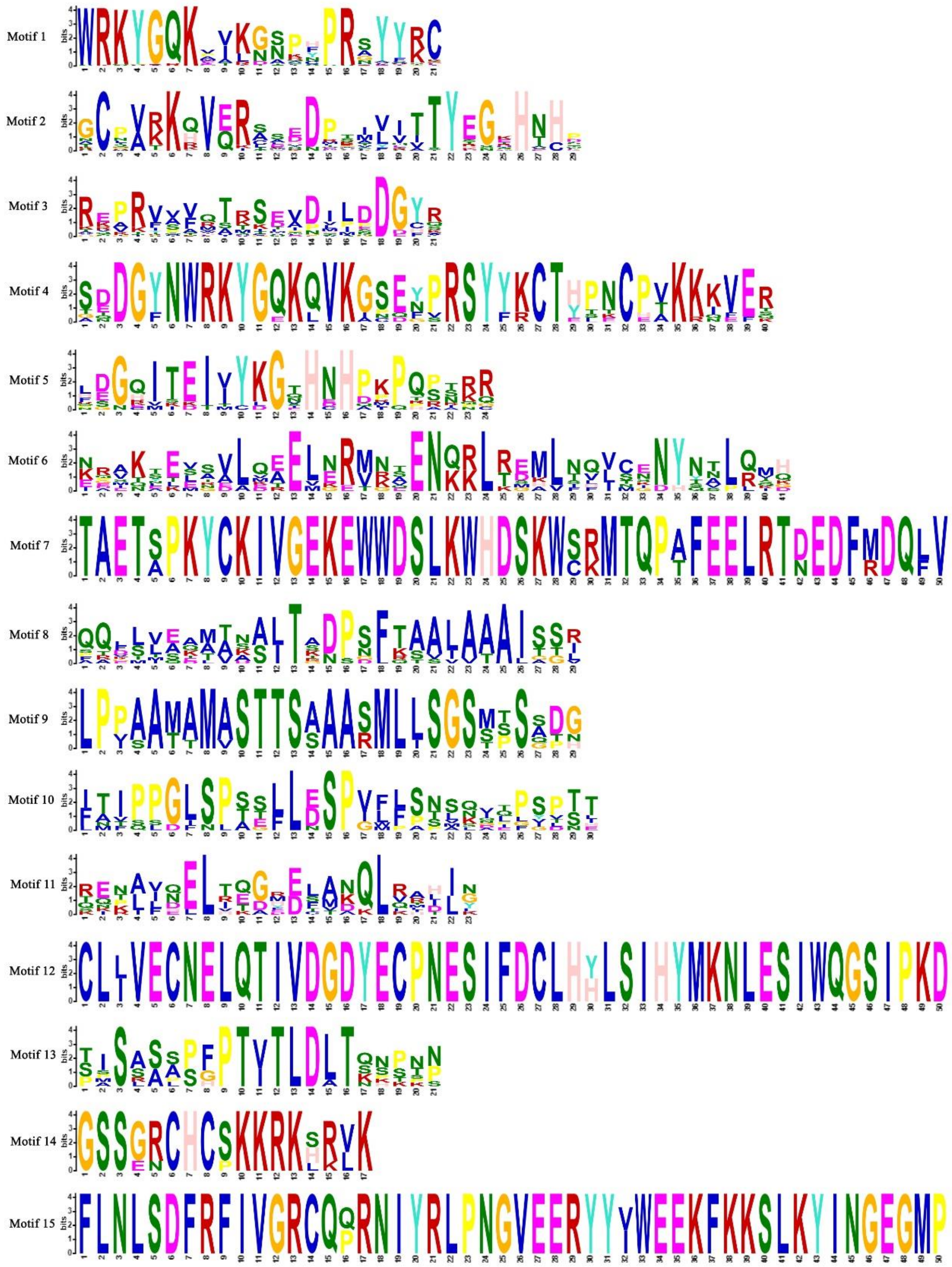

Figure S1. The specific sequence information of 15 motifs. The letters represent amino acids, the color does not represent any meaning, which are automatically synthesized by the MEME, and the height of the letters represents the motifs conservation.
